# Polymorphism in genes encoding two fatty acid binding proteins increases risk of ischemic stroke in a Chinese Han population

**DOI:** 10.3389/fgene.2023.1056186

**Published:** 2023-04-06

**Authors:** Maolin Cao, Yifei Zhang, Dan Chen, Jiaju Zhong, Xiaoli Zhang, Ling Yang, Xue Li, Liang Fang, Beizhong Liu, Fang Gong, Chanjuan Zhou

**Affiliations:** ^1^ Central Laboratory, Yongchuan Hospital of Chongqing Medical University, Chongqing, China; ^2^ Chongqing Clinical Research Center for Geriatric Disease, Chongqing, China; ^3^ Department of Rehabilitation Medicine, Yongchuan Hospital of Chongqing Medical University, Chongqing, China; ^4^ Department of Microbiology, Yongchuan Hospital of Chongqing Medical University, Chongqing, China; ^5^ Department of Neurology, Yongchuan Hospital of Chongqing Medical University, Chongqing, China; ^6^ Key Laboratory of Laboratory Medical Diagnostics, Ministry of Education, Department of Laboratory Medicine, Chongqing Medical University, Chongqing, China; ^7^ Department of General Practice, Yongchuan Hospital of Chongqing Medical University, Chongqing, China

**Keywords:** fatty acid-binding protein 1 (FABP1), fatty acid-binding protein 2 (FABP2), single nucleotide polymorphism (SNPs), ischemic stroke (IS), susceptibility

## Abstract

**Background:** Dyslipidemia is an independent predictor of ischemic stroke (IS). Genetic variations in lipid-metabolism related genes may increase the risk of IS. Fatty acid-binding protein 1 (FABP1) and fatty acid-binding protein 2 (FABP2) are lipid chaperones responsible for lipid transport and metabolism. The present study aimed to determine the association between *FABP1* or *FABP2* and ischemic stroke.

**Methods:** A total of 251 participants were recruited composed of 138 patients with ischemic stroke and 113 healthy subjects. DNA was extracted from peripheral blood samples. The rs2241883 polymorphism in *FABP1* and rs1799883 polymorphism in *FABP2* were genotyped using polymerase chain reaction-restriction fragment length polymorphism. Generalized multifactor dimensionality reduction (GMDR) was used to find out the interaction combinations between two SNPs and environmental factors.

**Results:** The GA genotype of FABP2 rs1799883 increased susceptibility to ischemic stroke under overdominant inheritance model (*p* = 0.042). After adjusting for the risk factors of IS, it was associated with a significantly higher risk of IS in the codominant inheritance model (adjust OR = 3.431, 95%CI = 1.060–11.103, *p* = 0.04). The interactions of *FABP1* rs2241883 and *FABP2* rs1799883 were not associated with IS risk (*p* = 0.172). Moreover, interaction analysis of two genes (rs1799883 and rs2241883) and two environmental factors (smoking and alcohol consumption) was associated with an increased risk of IS (*p* = 0.011).

**Conclusion:** The GA genotype of FABP2 rs1799883, interactions between rs1799883, rs2241883 and smoking and alcohol consumption were associated with IS risk in Chinese Han populations.

## 1 Introduction

Stroke is a leading cause of mortality and long-term disability, especially in low- and middle-income countries (Ma et al., 2021). The economic burden of stroke remains severe (GBD, 2016 Stroke Collaborators, 2019; Ma et al., 2021). Strokes are clinically categorized as ischemic stroke (IS), intracerebral hemorrhage, or subarachnoid hemorrhage. Among these, IS accounts for 60%–90% of cases (Liu et al., 2020; Kleindorfer et al., 2021). IS a complex disease that involves multiple genetic and environmental risk factors (Wang et al., 2018; Dichgans et al., 2019). Lipids are the key triggers of atherosclerosis, and dysfunction of lipid metabolism and its related processes play a vital role in the process of IS development (Chow et al., 2020). Several studies reported that lipid-related genes, such as EH domain-binding protein 1 and apolipoprotein E, are associated with an increased risk of IS (Liu et al., 2022; Qiao et al., 2022). Their genetic variation can cause lipid and lipid mediator dysregulation, which contribute to IS pathophysiology and possible mechanisms (Kloska et al., 2020). Hence, it is important to identify susceptibility genes in patients with IS for early screening and individualized treatment.

Fatty acid-binding proteins (FABPs), as lipid chaperones, are small intracellular proteins mediators that absorb, store, and export lipids (Hotamisligil and Bernlohr, 2015). Recently, drugs or antibodies targeting FABPs have provided new insights for managing chronic metabolic diseases (Hotamisligil and Bernlohr, 2015; Huang et al., 2022). Baier et al. first described a common nucleotide transition from guanine (G) to adenine (A) at codon 54 in exon 2 of *FABP2* (Ala54Thr, rs1799883) (Baier et al., 1995), which was shown to increase fat oxidation and insulin resistance (Baier et al., 1995; Albala et al., 2004; Albala et al., 2007). Furthermore, Brouillette et al. reported the Thr94Ala of *FABP1* (rs2241883), which could influence obesity indices as well as the risk of exhibiting residual hypertriglyceridemia (Brouillette et al., 2004). As a result, both FABP1 and FABP2 are associated with diseases accompanied by dysregulated lipid levels, such as metabolic syndrome, non-alcoholic fatty liver disease, and type 2 diabetes (Liu et al., 2015; Zare-Feyzabadi et al., 2022). However, the association between these genes and the risk of IS in the Chinese Han population remains unclear.

In this study, we validated the *FABP1* and *FABP2* polymorphisms in IS patients. We aimed to investigate two SNPs (rs1799883 and rs2241883) in the Han population in southwest China. We analyzed the associations between two SNPs(rs2241883 and rs1799883) and the risk of IS, gene-gene, and gene-environment interaction analyse.

## 2 Materials and methods

### 2.1 Participants

This study retrospectively included 138 patients with IS and 113 healthy controls (HCs) between October 2019 and June 2022 at Yongchuan Hospital of Chongqing Medical University. All participants were of Chinese Han descent and lived in southwest China. This study was approved by Yongchuan Hospital of Chongqing Medical University Research Ethics Committee, China. We followed the ethical guidelines of the Declaration of Helsinki and obtained written informed consent from all participants in our study.

The inclusion criteria for IS comprised age >18 years, symptoms of new-onset neurological deficits diagnostic confirmation by neurological examination and brain computed tomography (CT) or magnetic resonance imaging (MRI) according to the guidelines (Powers et al., 2019). The exclusion criteria comprised intracerebral or post-infarction hemorrhage, a history of liver, kidney, or autoimmune diseases, pregnant or breastfeeding women, or incomplete clinical data. Healthy persons were recruited from the Physical Examination Center of Yongchuan Hospital of Chongqing Medical University according to the following inclusion criteria: age >40 years, normal blood lipid and blood glucose values and liver and kidney functions.

### 2.2 Clinical evaluation

General information, including sociodemographic data, lifestyle characteristics, medical history, and laboratory data of all participants, was collected through face-to-face interviews or extracted from their medical records. Smoking was defined as at least one cigarette per day for half a year before admission, and alcohol consumption was defined as drinking any type of alcoholic beverage at least once a week for more than 6 months before admission. Serum total cholesterol (TC), hypertriglyceridemia (TG), low-density lipoprotein cholesterol (LDL-C), high-density lipoprotein cholesterol (HDL-C), apolipoprotein (Apo) A1, Apo B, aspartate aminotransferase (AST), alanine aminotransferase (ALT), total bilirubin (TBIL), direct bilirubin (DBIL), albumin (ALB), total protein (TB), fasting blood glucose, creatinine, white blood cell (WBC), red blood cell (RBC), hemoglobin (Hb), and platelets (PLT) levels were measured using standard laboratory methods.

### 2.3 DNA extraction and genotyping

Blood was obtained from all participants using an anticoagulant tube containing EDTA and quickly frozen at −80°C until use. Genomic DNA was extracted from the blood samples using TIANamp Blood DNA Kit (Tiangen Biotech, Beijing, China) according to the manufacturer’s protocol. The DNA samples were quantified using a NanoDrop-2000 instrument (Thermo Fisher Scientific, USA). All samples were genotyped in duplicate.

DNA nucleic acid PCR amplification was performed using a TaKaRa PCR Amplification Kit (Takara Bio, Japan) according to the manufacturer’s protocol. Briefly, DNA samples (0.5 μg) were amplified in a 50 μL volume consisting of 5 μL 10x PCR Buffer (1x), 4 μL dNTP mix (200 μM), 0.25 μL Taq enzyme (1.25 U),0.5 μL forward primer (20 μM), 0.5 μL reverse primer (20 μM) and 39.75 μL nuclease-free water. The primers for *FABP1* were: forward primer, 5′-CAG​TTG​GAA​GGT​GAC​AAT​AAA​CTG​TGA​CA-3′; reverse primer, 5′-GAG​GGG​TGG​CAT​TAG​GGT​ATG​TGA​G-3′. The primers of *FABP2* were: forward primer, 5′-ACA​GGT​GTT​AAT​ATA​GTG​AAA​AG-3′; reverse primer, 5′-TAC​CCT​GAG​TTC​AGT​TCC​GTC-3′. The PCR products obtained were analyzed on a 2% agarose gel stained with 4S Green Nucleic Acid Stain (Sangon Biotech, Shanghai, China) to certify amplification.

**FIGURE 1:**
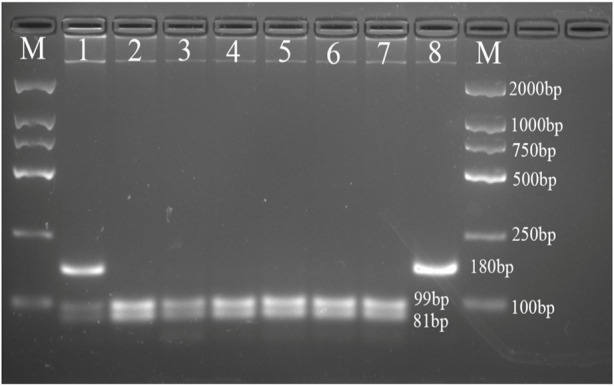
Agarose gel picture showing digested PCR products for FABP2 gene polymorphism. M, marker; 1 shows Ala/Thr genotype; 2, 3, 4, 5, 6, and 7 show Ala/Ala genotype; 8 shows Thr/Thr genotype.

**FIGURE 2:**
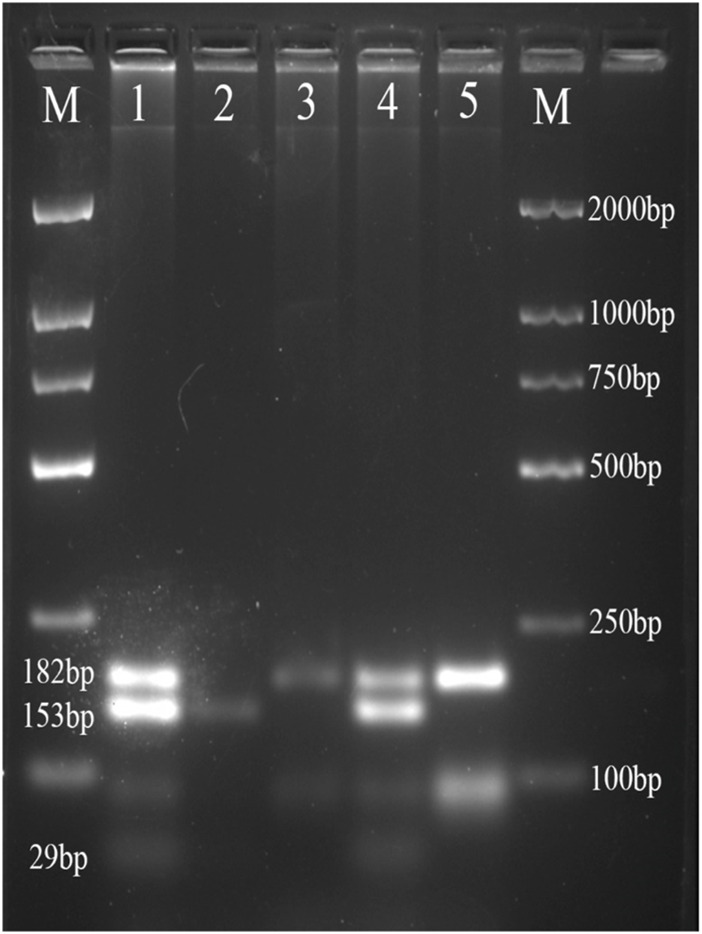
Agarose gel picture showing digested PCR products for FABP1 gene polymorphism. M, marker; 1 and 4 show Thr/Ala genotype; 2 shows Ala/Ala genotype; 3 and 5 shows Thr/Thr genotype.

The PCR products of *FABP1* were digested by Hind III endonuclease (Takara Bio, Japan), and PCR products of *FABP2* were digested by Hha I endonuclease (Takara Bio, Japan) according to the manufacturer’s protocol. Digestion products were electrophoresed on a 4% agarose gel and treated with 4S Green Nucleic Acid Stain. The bands were detected using the ChemiDoc XRS + System (Bio-Rad, USA). We detected the following FABP2 genotypes: Ala/Ala genotype with 2 fragments at 99 and 81bp; Ala/Thr genotype with three fragments at 180, 99, and 81bp; and Thr/Thr genotype with one 180bp fragment (Figure 1). Moreover, the FABP1 Thr/Thr genotype corresponded to 182bp; Thr/Ala genotype with three fragments at 182, 153, and 29bp, whereas Ala/Ala genotype was characterized by and two fragments of 153 and 29bp (Figure 2).

### 2.4 Statistical analyses

Data were analyzed using the Kolmogorov Smirnov test to determine their distributions. Normally distributed variables are presented as mean standard deviation (SD) and compared using the Student’s t-test. Non-normally distributed variables are expressed as median (interquartile range), and differences between groups were calculated using the Mann-Whitney U test. Categorical variables are presented as numbers (percentages) and were analyzed using the χ2 test or Fisher’s exact test. The Hardy Weinberg equilibrium (HWE) test of genotype distribution was performed using the χ2 test for goodness of fit. Logistic regression analysis was performed to determine the association between genetic polymorphisms and the risk of stroke and other risk factors. Gene-gene and gene-environmental interactions were analyzed using generalized multifactor dimensionality reduction (GMDR). Statistical significance was set at *p* < 0.05. Statistical analyses were performed using the SPSS version 25.0 software (IBM SPSS Inc., USA) and GMDR software version 09 (http://sourceforge.net/projects/gmdr/). The sample size was caculated by Quanto 1.2.4 software (https://quanto.software.informer.com/download/#downloading) with the following parameters: the prevalence of ischemic stroke was 1,255.9 per 100,000 population (Wang et al., 2022), the allele frequency of the FABP1 genes and the FABP2 genes in the East Asian was 0.2136 and 0.685 in the National Center of Biotechnology Information (NCBI) dbSNP database, α was 0.05, 1-β was 0.8, and expected Odd Rate value was 2–4 according to previous studies (Yamada et al., 2008; Mansego et al., 2012; Peng et al., 2012; Garcés Da Silva et al., 2018). The predicted results showed that 35 to 133 and 161 to 484 samples were needed for the FABP1 and FABP2 gene, respectively.

## 3 Results

### 3.1 Characteristics of study population

The baseline characteristics of the 251 participants are presented in Table 1. There were 138 people in the IS group and 113 people in the HCs group. The mean age of all participants was 63.6 ± 13.7 years, and 53% were male. Patients with IS had a higher prevalence of hypertension, diabetes, dyslipidemia, coronary artery disease, atrial fibrillation, smoking, and alcohol consumption (*p* < 0.05). Compared to the HCs, the patients with IS were older, and had higher blood pressure, AST, ALT, DBIL, fasting blood glucose, and WBC (*p* < 0.05). However, the level of ALB, creatinine, RBC, and Hb were lower than those in HCs. There were no significant differences in the levels of TBIL, TB, or PLT between two groups (*p* > 0.05). For the blood lipid testing, the plasma levels of Apo B were higher in patients with IS (*p* < 0.05), while the levels of TC, HDL-C, and LDL-C were lower in patients with IS (*p* < 0.05). No difference of TG level was found (*p* > 0.05).

**TABLE 1:** Characteristics of the participants.

		Ischemic stroke (n = 138)	Healthy control (n = 113)	*p*-Value
Sex (%)	female	57 (41.3)	61 (54.0)	0.045
	male	81 (58.7)	52 (46.0)	
Age (years)^b^		70.0 ± 12.2	55.7 ± 11.2	<0.001
Smoke (%)		48 (34.8)	0 (0.0)	<0.001
Alcohol consumption (%)		50 (36.2)	20 (17.7)	0.001
Systolic blood pressure (mmHg)^a^		145 (127,165)	121 (110,133)	<0.001
Diastolic blood pressure (mmHg)^b^		86.5 ± 16.4	71.7 ± 9.7	<0.001
History	Hypertension (%)	91 (65.9)	11 (9.7)	<0.001
	Diabetes (%)	36 (14.3)	0 (0.0)	<0.001
	Dyslipidemia (%)	16 (11.6)	0 (0.0)	<0.001
	Coronary artery disease (%)	39 (28.3)	1 (0.9)	<0.001
	Atrial fibrillation (%)	27 (19.6)	0 (0.0)	<0.001
	Stroke (%)	21 (15.2)	1 (0.9)	<0.001
Blood lipids	TC (mmol/L)^a^	4.3 (3.5,4.9)	4.5 (4.2,4.8)	0.022
	TG (mmol/L)^a^	1.2 (0.84,1.7)	1.1 (0.8,1.4)	0.141
	HDL-C (mmol/L)^b^	1.3 ± 0.3	1.5 ± 0.3	<0.001
	LDL-C (mmol/L)^a^	2.3 (1.8,3.0)	2.6 (2.2,2.8)	0.039
	Apo A1 (mmol/L)^b^	1.2 ± 0.2	1.5 ± 0.2	<0.001
	Apo B (mmol/L)^a^	0.9 (0.7,1.0)	0.8 (0.7,0.9)	0.004
Liver function^a^	ALT (U/L)	27.0 (18.0,38.3)	16.0 (12.0,26.0)	<0.001
	AST (U/L)	27.5 (21.0,36.3)	18.0 (15.0,21.0)	<0.001
	TBIL (umol/L)	10.2 (7.6,14.3)	10.2 (8.1,14.0)	0.537
	DBIL (umol/L)	2.6 (1.7,3.8)	2.2 (1.7,2.8)	0.02
	TB (g/L)	68.9 (63.1,74.3)	69.6 (67.5,71.9)	0.285
	ALB (g/L)	38.9 (35.0,41.4)	44.3 (42.4,46.4)	<0.001
Creatinine (umol/L)^a^		61.5 (52.8,76.3)	72.0 (59.5,84.0)	<0.001
Glucose (mmol/L)^a^		6.2 (5.1,7.8)	5.4 (5.1,5.7)	<0.001
WBC(×10^9/L)^a^		7.3 (6.0,8.9)	5.6 (4.9,6.3)	<0.001
RBC(×10^12/L)^a^		4.3 (3.9,4.7)	4.6 (4.2,5.0)	<0.001
Hb(g/L)^a^		131.0 (118.0.143.0)	139.0 (128.5,153.0)	<0.001
PLT (×10^9/L)^a^		189.0 (150.8,232.5)	210.0 (168.0.253.5)	0.052
TOAST classification	large-artery atherosclerosis (%)	49 (35.5)		
	cardioembolism (%)	37 (26.8)		
	small-vessel occlusion (%)	18 (13.0)		
	stroke of other determined etiology (%)	28 (20.3)		
	stroke of undetermined etiology (%)	6 (4.3)		
NHISS score^a^		11.5 (6.0,17.0)		
GCS score^a^		14.0 (10.0,15.0)		
mRS score^a^		4.0 (3.0,5.0)		
Statins (%)		5 (3.6)		

^a^ Median (Q1, Q3).

^b^ Mean (SD).

### 3.2 Genotype frequency distribution of SNPs and mutation frequency of alleles

The genotype frequency distributions of SNPs in HWE and mutation frequencies of the alleles are shown in Table 2.

**TABLE 2:** Genotype frequency and Hardy-Weinberg equilibrium distribution of SNPs and mutation frequency of alleles.

Genotype			Ischemic stroke (n = 138)	Healthy control (n = 113)	*p*-Value
FABP2 2445G→A (Ala54Thr)	Codominant	GG	71 (51.4)	70 (61.9)	0.122
		GA	60 (43.5)	35 (31.0)	
		AA	7 (5.1)	8 (7.1)	
	P_HWE_ value		0.205	0.227	
	Dominant	GG	71 (51.4)	70 (61.9)	0.095
		GA + AA	67 (48.6)	43 (38.1)	
	Recessive	AA	7 (5.1)	8 (7.1)	0.505
		GG + GA	131 (95.0)	105 (92.9)	
	Overdominant	GA	60 (43.5)	35 (31.0)	0.042
		GG + AA	78 (56.6)	78 (69.0)	
	Allele frequency	G	202 (73.2)	175 (77.4)	0.274
		A	74 (26.8)	51 (22.6)	
FABP1 A→G (Thr94Ala)	Codominant	AA	66 (47.8)	41 (36.3)	0.148
		AG	66 (47.8)	68 (60.2)	
		GG	6 (4.3)	4 (3.5)	
	P_HWE_ value		0.035	<0.001	
	Dominant	AA	66 (47.8)	41 (36.3)	0.066
		AG + GG	72 (52.2)	72 (63.7)	
	Recessive	GG	6 (4.3)	4 (3.5)	0.745
		AA + AG	132 (95.7)	109 (96.5)	
	Overdominant	AG	66 (47.8)	68 (60.2)	0.086
		AA + GG	45 (40.5)	72 (51.4)	
	Allele frequency	A	198 (71.7)	150 (66.4)	0.194
		G	78 (28.3)	76 (33.6)	

P_HWE_, Hardy-Weinberg equilibrium test value.

FABP2 re1799883: In the IS group, FABP2 genotype frequencies were GG (51.4%), GA (43.5%), and AA (5.1%), and those in HCs were 61.9%, 31%, and 7.1%, respectively. In the overdominant inheritance model (GG + AA vs GA), the GA frequency of *FABP2* rs1799883 in IS patients was significantly higher than that in HCs (*p* = 0.042). No significant difference has not been reached for codominant, dominant, or recessive inheritance model.

FABP1 rs2241883: FABP1 genotype frequencies were AA (47.8%), AG (47.8%), and GG (4.3%), and those in HCs were 36.3%, 60.2%, and 3.5%, respectively. The rs2241883 polymorphism did not differ between IS patients and HCs under all genotype models (*p* > 0.05).

### 3.3 Association analysis between SNPs and risk of IS

Logistic regression analysis was performed to screen the risk for associations between candidate SNPs and IS risk (Table 3). Neither *FABP2* rs1799883 nor *FABP1* rs2241883 of the *FABP1* in four different inheritance models had significant effect on the risk of IS (*p* > 0.05). After using multivariable logistic recessive adjusted risk factors for IS, we found that patients with the FABP2 rs1799883 GA genotype had a significantly higher risk of stroke in the codominant inheritance model (adjusted odds ratio (OR):3.431; 95%Confidence interval (CI):1.060–11.103; *p* = 0.04).

**TABLE 3:** Association analysis between SNPs and risk of ischemic stroke.

Genotype			OR (95%CI)	*p*-Value	Adjust OR (95%CI)^a^	*p*-Value
FABP2 2445G→A (Ala54Thr)	Codominant	GG	ref		ref	
		GA	1.690 (0.993–2.876)	0.053	3.431 (1.060–11.103)	0.040
		AA	0.863 (0.297–2.507)	0.786	0.314 (0.026–3.838)	0.365
	Dominant	GG	ref		ref	
		GA + AA	1.426 (0.862–2.359)	0.167	2.120 (0.720–6.246)	0.173
	Recessive	AA	ref		ref	
		GG + GA	1.426 (0.501–4.060)	0.506	5.550 (0.503–61.193)	0.162
	Overdominant	GA	ref		ref	
		GG + AA	0.601 (0.356–1.013)	0.056	0.331 (0.107–1.027)	0.056
FABP1 A→G (Thr94Ala)	Codominant	AA	ref		ref	
		AG	0.603 (0.360–1.010)	0.055	0.518 (0.180–1.489)	0.222
		GG	0.932 (0.248–3.502)	0.917	2.264 (0.210–24.439)	0.501
	Dominant	AA	ref		ref	
		AG + GG	0.21 (0.374–1.033)	0.067	0.590 (0.210–1.654)	0.316
	Recessive	GG	ref		ref	
		AA + AG	0.807 (0.222–2.934)	0.745	0.311 (0.031–3.080)	0.318
	Overdominant	AG	ref		ref	
		AA + GG	1.648 (0.996,2.727)	0.052	2.102 (0.756–5.847)	0.155

OR, odds ratio; 95% CI, 95% confidence interval.

^a^ adjust risk factors of stroke, including sex, age, smoke, systolic blood pressure, diastolic blood pressure, hypertension, diabetes, dyslipidemia, coronary artery disease, atrial fibrillation, stroke, TC, TG, HDL-C, and LDL-C.

### 3.4 Association of various genotypes with risk factors for IS

To evaluate the association between genotype polymorphisms and risk factors for IS, we found that patients with ischemic stroke who carried GG in *FABP2* had a higher prevalence of atrial fibrillation (*p* = 0.032), and those patients who carried AG in *FABP1* were older than those with other genotypes (*p* = 0.048). There were no significant statistical differences in sex, blood pressure, hypertension, diabetes, dyslipidemia, coronary artery disease, stroke, and blood lipid levels in both FABP2 (Table 4) and FABP1 (Table 5) genotypes.

**TABLE 4:** Association between different FABP2 genotype and risk factors for ischemic stroke.

Genotype		Ischemic stroke (n = 138)			*p*-Value	Healthy control (n = 113)			*p*-Value
		GG (n = 71)	GA (n = 60)	AA (n = 7)		GG (n = 70)	GA (n = 35)	AA (n = 8)	
Sex (%)	Female	26 (36.6)	28 (46.7)	3 (42.9)	0.519	41 (58.6)	16 (45.7)	4 (50.0)	0.429
	Male	45 (63.4)	32 (35.2)	4 (57.1)		29 (41.4)	19 (54.3)	4 (50.0)	
Age (years)^a^		72.5 ± 6.4	57 ± 6.9	79.0 ± 0.0	0.373	57.0 ± 9.3	68.0 ± 12.7	47.0 ± 0.0	0.433
Smoking (%)		28 (39.4)	18 (30.0)	4 (57.1)	0.258	11 (15.7)	8 (22.9)	1 (12.5)	0.614
Systolic blood pressure (mmHg)^a^		156.0 ± 25.5	161.0 ± 15.9	155.0 ± 0.0	0.247	116.1 ± 9.8	102.5 ± 9.2	96.0 ± 0.0	0.445
Diastolic blood pressure (mmHg)^a^		78.3 ± 10.8	95.0 ± 8.6	122.0 ± 0.0	0.321	66.6 ± 8.8	58.0 ± 7.1	58.0 ± 0.0	0.320
History	Hypertension (%)	49 (69.0)	35 (58.3)	7 (100.0)	0.062	6 (8.6)	4 (11.4)	1 (12.5)	0.678
	Diabetes (%)	19 (26.8)	14 (23.3)	3 (42.9)	0.49	0 (0.0)	0 (0.0)	0 (0.0)	-
	Dyslipidemia (%)	9 (12.7)	6 (10.0)	1 (14.3)	0.747	0 (0.0)	0 (0.0)	0 (0.0)	-
	Coronary artery disease (%)	24 (33.8)	14 (23.3)	1 (14.3)	0.364	1 (1.4)	0 (0.0)	0 (0.0)	1.000
	Atrial fibrillation (%)	51 (28.2)	7 (11.7)	0 (0.0)	0.032	0 (0.0)	0 (0.0)	0 (0.0)	-
	Stroke (%)	12 (16.9)	8 (13.3)	1 (14.3)	0.85	0 (0.0)	1 (2.9)	0 (0.0)	0.381
Blood lipids	TC (mmol/L)^a^	4.4 ± 1.0	4.3 ± 1.3	3.7 ± 1.1	0.27	4.4 ± 0.5	4.6 ± 0.5	4.7 ± 0.2	0.095
	TG (mmol/L)^a^	1.4 ± 0.7	1.4 ± 1.0	1.1 ± 0.3	0.635	1.1 (0.8,1.4)	1.1 (0.8,1.5)	1.1 (0.9,1.5)	0.916
	HDL-C (mmol/L)^a^	1.3 ± 0.3	1.3 ± 0.3	1.1 ± 0.2	0.171	1.4 ± 0.3	1.5 ± 0.2	1.5 ± 0.1	0.464
	LDL-C (mmol/L)^a^	2.5 ± 0.9	2.4 ± 1.0	2.0 ± 0.7	0.381	2.5 ± 0.5	2.6 ± 0.4	2.8 ± 0.3	0.154

^a^ Mean (SD).

**TABLE 5:** Association between different FABP1 genotype and risk factors for ischemic stroke.

		Ischemic stroke (n = 138)			*p*-Value	Healthy control (n = 113)			*p*-Value
		AA (n = 66)	AG (n = 66)	GG (n = 6)		AA (n = 41)	AG (n = 68)	GG (n = 4)	
Sex (%)	Female	23 (34.8)	30 (45.5)	4 (66.7)	0.217	22 (53.7)	37 (54.4)	2 (50.0)	1.000
	Male	43 (65.2)	36 (54.5)	2 (33.3)		19 (46.3)	31 (45.6)	2 (50.0)	
Age (years)^b^		67.9 ± 11.9	72.8 ± 12.0	62.7 ± 11.9	0.048	58.1 ± 11.9	54.7 ± 10.4	48.0 ± 13.5	0.115
Smoking (%)		27 (40.9)	23 (34.8)	0 (0.0)	0.13	8 (19.5)	11 (16.2)	1 (25.0)	0.741
Systolic blood pressure (mmHg)^b^		150.2 ± 31.1	150.9 ± 28.4	130.0 ± 18.1	0.247	124.1 ± 17.9	121.7 ± 17.5	132.5 ± 26.2	0.45
Diastolic blood pressure (mmHg)^b^		86.9 ± 15.9	87.2 ± 17.3	76.7 ± 7.3	0.321	70.4 ± 10.0	72.2 ± 17.5	77.8 ± 6.9	0.303
History	Hypertension (%)	40 (60.6)	46 (69.7)	5 (83.3)	0.401	5 (12.2)	6 (8.8)	0 (0.0)	0.831
	Diabetes (%)	19 (28.8)	17 (25.8)	0 (0.0)	0.449	0 (0.0)	0 (0.0)	0 (0.0)	-
	Dyslipidemia (%)	4 (6.1)	11 (16.7)	1 (16.7)	0.151	0 (0.0)	0 (0.0)	0 (0.0)	-
	Coronary artery disease (%)	16 (24.2)	22 (33.3)	1 (16.7)	0.514	1 (2.4)	0 (0.0)	0 (0.0)	0.398
	Atrial fibrillation (%)	9 (13.6)	17 (25.8)	1 (16.7)	0.235	0 (0.0)	0 (0.0)	0 (0.0)	-
	Stroke (%)	10 (15.2)	11 (16.7)	0 ((0.0)	0.553	1 (2.4)	0 (0.0)	0 (0.0)	0.398
Blood lipids	TC (mmol/L)^b^	4.3 ± 1.0	4.4 ± 1.3	3.7 ± 1.1	0.324	4.5 ± 0.5	4.5 ± 0.4	4.5 ± 0.5	0.858
	TG (mmol/L)^a^	1.2 (0.9,1.7)	1.2 (0.8,1.6)	1.3 (0.9,1.8)	0.545	0.9 (0.8,1.4)	1.1 (0.8,1.4)	1.2 (0.8,1.9)	0.342
	HDL-C (mmol/L)^b^	1.3 ± 0.3	1.3 ± 0.3	1.2 ± 0.3	0.415	1.5 ± 0.2	1.4 ± 0.3	1.5 ± 0.4	0.591
	LDL-C (mmol/L)^b^	2.4 ± 0.8	2.5 ± 1.1	2.0 ± 0.9	0.397	2.5 ± 0.5	2.5 ± 0.4	2.5 ± 0.3	0.984

^a^ Median (Q1, Q3)

^b^ Mean (SD).

### 3.5 Gene-gene and gene-environment interactions on IS

**FIGURE 3:**
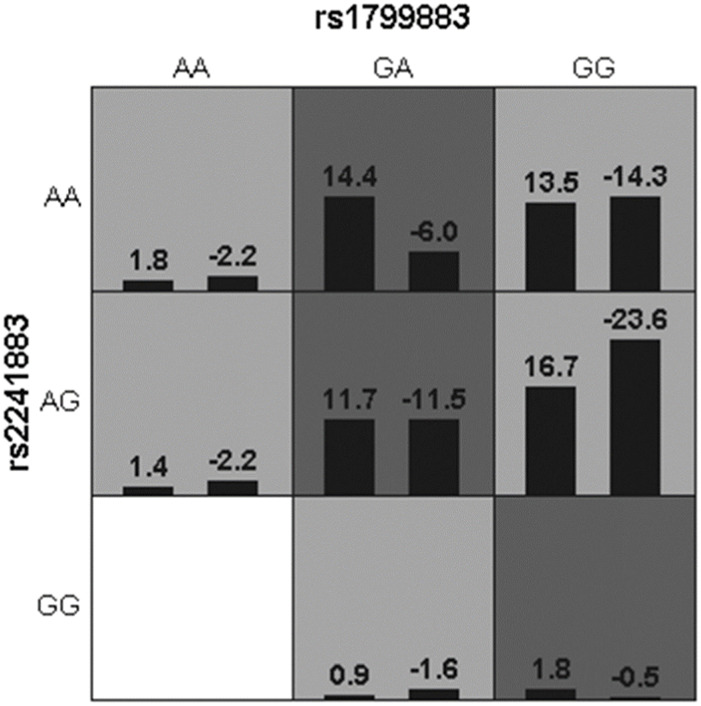
The optimal interaction model of rs1799883 and rs22241883.

**FIGURE 4:**
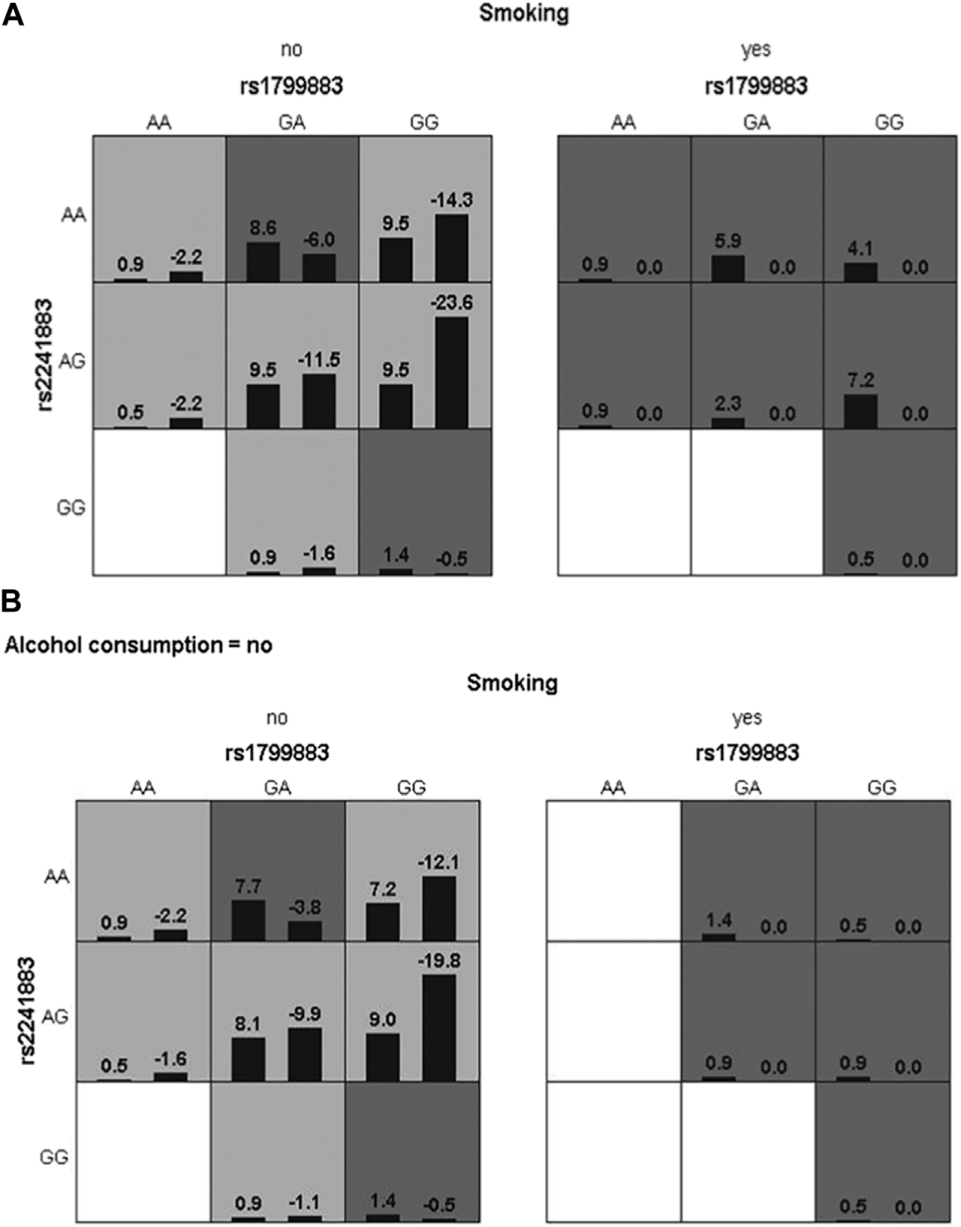
The optimal interaction model of genes and environmental factors. **(A)** Interaction between two genes and smoking. **(B)** interaction between two genes and smoking and alcohol consumption.

Lastly, gene-gene and gene-environment interactions were analyzed to further explore genotype polymorphisms and environmental impact in the risk of IS. As shown in Table 6 and Figure 3, the rs1799883-rs2241883 interaction was not associated with IS risk. In the gene-environment interaction analysis, rs1799883, rs2241883, and smoking and alcohol consumption were correlated with an increased risk of IS (*p* = 0.011), and the model had good cross-validation consistency and training balance accuracy (Table 6). The optimal interaction model of gene-gene and gene-environment interactions is shown in Figure 3 and Figure 4.

**TABLE 6:** Gene-gene and gene-environment interaction on ischemic stroke.

	Training balance	Testing balance	*p*-Value	CV consistency
Model 1				
rs1799883	0.5686	0.5049	0.623	6/10
rs1799883, rs2241883	0.5834	0.5077	0.172	10/10
Model 2				
rs1799883, Smoke	0.6783	0.626	0.055	5/10
rs1799883, rs2241883, Smoke	0.7016	0.6595	0.011	8/10
rs1799883, rs2241883, Smoke, Alcohol consumption	0.7138	0.6625	0.011	10/10

CV, cross-validation; Model 1, gene-gene interaction on ischemic stroke; Model 2, gene-environment interaction on ischemic stroke.

## 4 Discussion

Lipids play a fundamental role in the pathogenesis atherosclerosis, which can contribute to disease development and mortality, including IS. As lipid chaperones, FABPs mediate both lipid-related biological processes and systemic metabolic homeostasis by regulating multiple lipid signals (Furuhashi and Hotamisligil, 2008). Therefore, understanding the relationship between FABPs and ischemic stroke is important. Here we evaluated the associations between alleles of FABP1 and FABP2 genes and ischemic stroke risk in 251 Chinese Han patients.

We uncovered significantly different genotype frequencies of FABP2 in the overdominant inheritance model compared to those in HCs. The Ala54Thr polymorphism of *FABP2* is associated with metabolic or cardiovascular diseases (Wanby et al., 2005; Abbas et al., 2015; Liu et al., 2015). Similar to Panby’s work (Wanby et al., 2007), we found that patients carrying FABP2 rs1799883 GA genotype might have increased IS risk. This indicated that the Ala54 site may be a risk predictor for IS in Han population. The FABP2 rs1799883 is a common SNP at codon 54 in exon 2(Ala54Thr) located at Chr4q28-q31 (Furuhashi and Hotamisligil, 2008), which could lead to changed gene expression and biofunctions of FABP2(Formanack and Baier, 2004). Compared to the alanine (Ala)-containing protein in FABP2, the threonine (Thr)-containing protein has a twofold higher binding affinity for long-chain fatty acids in human (Baier et al., 1995). An vitro study also showed that Thr54 mutation enabled FABP2 to transport more long-chain fatty acids, resulting in enhanced triglycerides secrete (Baier et al., 1996). Besides, the Ala54Thr polymorphism of *FABP2* could increase the small intestinal lipid absorption (Levy et al., 2001). Increasing evidence has observed the changes in blood fatty acid metabolisms in patients with acute IS (Au, 2018; Wang et al., 2020). Therefore, one possible explanation of our findings is that people carrying rs1799883 GA genotype may have dysregulated fatty acids metabolisms, which contribute to the high risk of IS. As we did not measure the fatty acid levels in patients with ischemic stroke, the relationship between fatty acid levels and the FABP2 Ala54Thr genotype requires further validation. Notably, the present findings of the relationship between Ala54Thr polymorphism and IS differ from those of an earlier study (Yamada et al., 2008). Ischemic stroke is a complex disease caused by various genetic and environmental factors. Different diets, geographical environments, and genetic backgrounds can lead to altered susceptibilities.

The Thr94Ala of FABP1 is a polymorphism at condon 94 in exon 3 at Chr2p12-q11. One study reported that FABP1 Thr94Ala variant affected TG accumulation in human hepatocytes (McIntosh et al., 2014). Although *FABP1* Thr94Ala polymorphisms are also likely related to influence plasma lipid levels (Fisher et al., 2007; Tian et al., 2015; Valizadeh et al., 2021), we found that blood lipid levels were not related to stroke risk under all genotype models. We did not find an association between *FABP1* Thr94Ala and IS either, suggesting that the Thr94Ala polymorphism of *FABP1* may not be an independent risk factor for IS in this Han population. The Thr94Ala polymorphism in FABP1 is a susceptibility site for atherothrombotic cerebral infarction among Japanese patients with metabolic syndrome (Yamada et al., 2008). However, we included all types of IS rather than IS patients who were diagnosed with metabolic syndrome before, pervious study might overestimation risk of stroke. In addition, we found that genotype frequencies of FABP1 deviate from HWE in HCs. Since selection, mutations, linkage disequilibrium, population stratification and inbreeding could cause deviations from HWE in a population (Chen et al., 2009), it would be difficult for us to find out the potential origins of the FABP1 deviation in the included populations. Lager cohort study may need to be further validated.

We assessed the impact of *FABP1-FABP2*, and gene-environmental interaction on IS risk. Genetic and environmental factors, as well as gene-gene and gene-environment interactions, are related to the incidence of IS (Ferreira et al., 2019; Zhang et al., 2019). In our study, *FABP1*-*FABP2* interactions did not contribute to IS risk, indicating that *FABP1* and *FABP2* interaction cannot adequately explain interindividual variability in patients with IS. Notably, the interaction between two FABPs (rs2241883 in *FABP1* and rs1799883 in *FABP2*) and two environmental factors (smoking and alcohol consumption) significantly increased the risk of IS. Smoking and alcohol consumption have previously been shown to increase the risk of IS (Ma et al., 2021; Roerecke, 2021). Thus, patients carrying *FABP1* rs2241883 or *FABP2* rs17799883 should reduce smoking and alcohol consumption to reduce the risk of IS. In addition, our study also indicated that *FABP1* and *FABP2* polymorphisms can be potential early biomarkers for IS risk, providing crucial information for identifying its underlying mechanisms.

Several limitations should be addressed in our study. Firstly, although the sample was small. We observed the associations between FABP2 rs1799883 polymorphisms and ischemic stroke. Nonetheless studies with larger sample sizes form different ethnicities are helpful to confirm our findings. Secondly, numerous environmental factors are associated with ischemic stroke. Yet, owing to limited clinical data, only the two most important environmental risk factors were analyzed. Thirdly, although we discovered potential interactions between genes and environmental conditions for IS, the connecting mechanisms between stroke risk, smoking, and alcohol consumption remain unclear. Finally, etither FABP1 or FABP2 protein involves in the absorption and transportation of lipids. As a result, further studies are needed to identify the impacts of FABPs’ gene polymorphisms on lipid-related metabolites. This will facilitate a better understanding of the relationship among FABP polymorphisms, lipid metabolism, and the IS risk.

## 5 Conclusion

This study examined the associations of *FABP1* and *FABP2*, gene-gene, and gene-environment, with IS in a Han population in Southwest China. Rs2241883 in *FABP1* and rs1799883 in *FABP2* were not associated with IS. After adjusting for risk factors, the GA genotype of FABP2 might increase the risk of IS in the codominant inheritance model. Our results indicate that gene-environment interactions may increase IS risk.

## Data Availability

The original contributions presented in the study are included in the article/Supplementary Material, further inquiries can be directed to the corresponding authors.
